# Cholesterol: A Gatekeeper of Male Fertility?

**DOI:** 10.3389/fendo.2018.00369

**Published:** 2018-07-19

**Authors:** Lauriane Sèdes, Laura Thirouard, Salwan Maqdasy, Manon Garcia, Françoise Caira, Jean-Marc A. Lobaccaro, Claude Beaudoin, David H. Volle

**Affiliations:** Université Clermont Auvergne, INSERM U 1103, CNRS, UMR 6293, Génétique Reproduction et Développement, Clermont-Ferrand, France

**Keywords:** cholesterol, fertility, testis, spermatozoa, nuclear receptors

## Abstract

Cholesterol is essential for mammalian cell functions and integrity. It is an important structural component maintaining the permeability and fluidity of the cell membrane. The balance between synthesis and catabolism of cholesterol should be tightly regulated to ensure normal cellular processes. Male reproductive function has been demonstrated to be dependent on cholesterol homeostasis. Here we review data highlighting the impacts of cholesterol homeostasis on male fertility and the molecular mechanisms implicated through the signaling pathways of some nuclear receptors.

Infertility is a major public health issue defined by the World Health Organization as the inability of a couple to conceive a child after 1 year of unprotected regular sex. About 15% of couples worldwide are reported to be infertile (1); and male disorders are diagnosed in 30–50% of cases (2). Azoospermia is one of the most severe case of infertility; which is defined by the total absence of spermatozoa in the ejaculate and affects more than 20% of infertile men (3).

Male infertility can be divided into two categories: abnormalities of excretory origin, which define a defect of sperm delivery in the genital tract and abnormalities of secretory origin, which correspond an alteration of the production of the spermatozoa by the testis. The aetiologies of testicular lesions involve multiple genetic, environmental and/or behavioral factors. However, in 30% of cases, male fertility disorders remain unexplained (4). It is therefore necessary to better understand the physiology of the testis in order to identify the causes of infertility that are still idiopathic.

Male reproductive function has been demonstrated to be highly dependent on cholesterol homeostasis. Indeed, cholesterol is the precursor of steroid synthesis which is crucial for normal sperm production (5–7). Moreover, many experimental and clinical data have highlighted the importance of lipid metabolism in the control of testicular physiology and male fertility (8). As example, the depletion of cholesterol in plasma and tissues in a mouse model deficient for the gene encoding 24-dehydrocholesterol reductase (*Dhcr24*) leads to mouse infertility (9).

## Cholesterol homeostasis

Cholesterol is essential for mammalian cell functions and integrity. It is an important structural component maintaining the permeability and fluidity of the cell membrane. Within the cells, the cholesterol homeostasis is tightly regulated to ensure normal cellular processes. Cholesterol homeostasis is strictly regulated at the cellular level.

Cholesterol is also a precursor for steroid hormones synthesis such as bile acids and vitamin D. Oxidized derivatives of cholesterol, known as oxysterols, are also implicated in numerous biological processes.

The main source of cholesterol is food. Dietary cholesterol is first routed from the small intestine to the liver, and then redistributed to the requesting organs. Cholesterol is uptake from lipoproteins; the Scavenger receptor B type 1 (SR-B1) is the cell surface high-density lipoprotein (HDL) receptor that mediates HDL-cholesterol ester (HDL-CE) uptake. Next to this, less than half of the cholesterol originates from *de novo* synthesis. Cholesterol synthesis is a multi-step enzymatic process. Synthesis of cholesterol begins from the acetyl-CoA, which is transported from the mitochondria to the cytosol. A series of reactions give rise to Hydroxy-methylglutaryl-CoA (HMG-CoA), which is then converted to mevalonate by HMG-CoA reductase. Finally, multi-steps will then give rise to cholesterol.

The cellular efflux of cholesterol is essential to maintain the homeostasis, as cells in peripheral organs do not express genes involved in the catabolism of cholesterol. The efflux of cholesterol requires acceptors such as HDL. There is either a simple diffusion (aqueous diffusion pathway) or a facilitated one through SR-BI pathway. There could also be active process *via* members of the ATP-binding cassette (ABC) family transporters such as ABCA1 and ABCG1.

**Figure 1:**
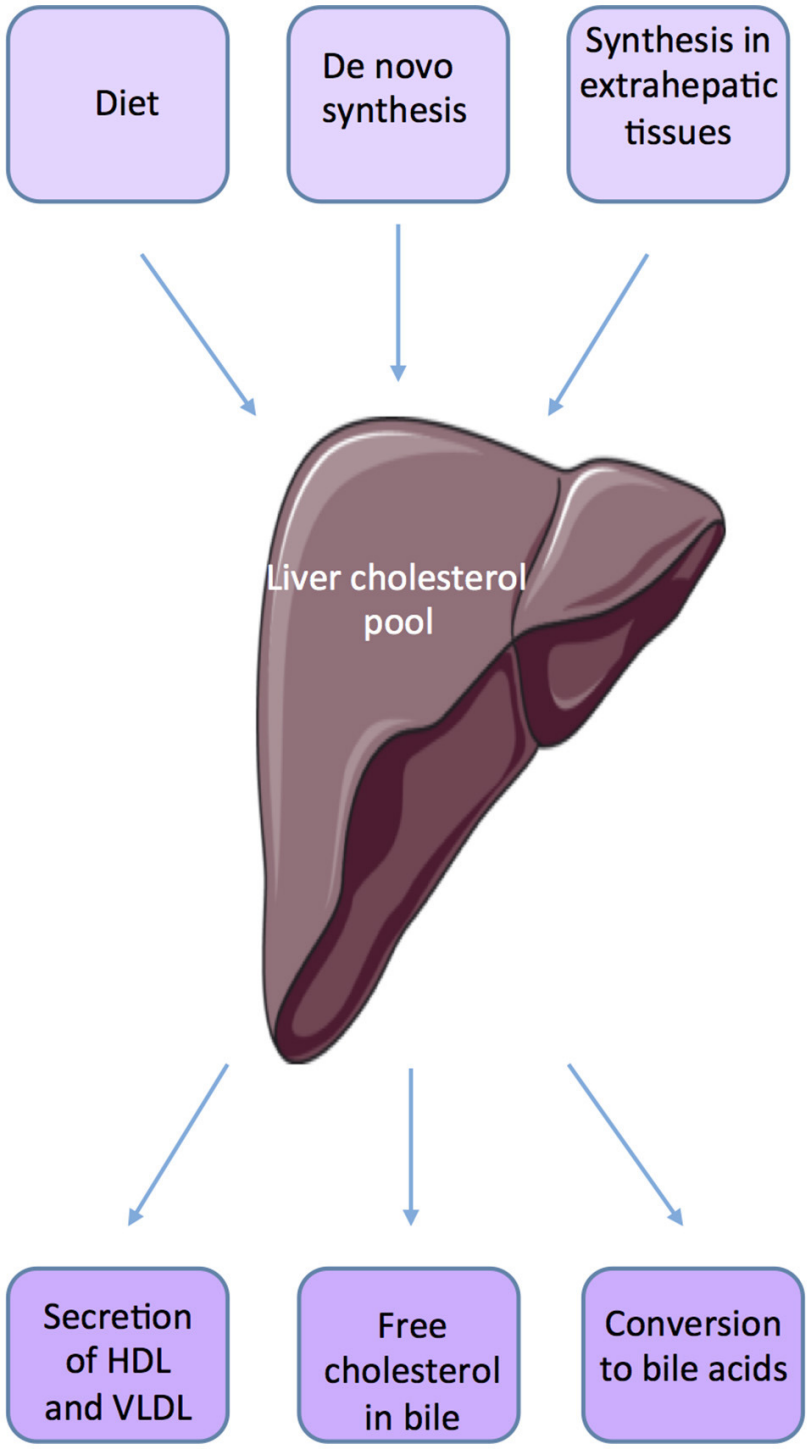
Source of cholesterol.

Within the body, the liver plays an important role in maintaining cholesterol homeostasis by regulating absorption and synthesis to prevent over accumulation in the plasma and tissues (10, 11) (Figure 1). The small intestine is also an important actor of *de novo* synthesis of cholesterol, bile synthesis, and absorption and fecal excretion. Molecular mechanisms involved have been quite well defined in the organs such as liver, adipose tissue, and intestine (12). The cross talks between liver and intestine illustrate the complex mechanisms involved in the regulation of cholesterol homeostasis through the involvement of several factors such as SREBP-2 (sterol regulatory element-binding protein), LXR (Liver X receptor, NR1H2, NR1H3), and FXRα (Farnesoid X receptor, NR1H4). Many studies suggest that SREBP and LXRs work in a coordinated manner to maintain cholesterol homeostasis (11, 13). SREBP2 up-regulates the expression of genes involved in the biosynthesis and uptake of cholesterol when cholesterol level is low.

LXRα and LXRβ are ligand-activated nuclear receptors; which are critical for the elimination of excess cholesterol. LXRα is primarily expressed in macrophages, intestine, liver, adipose, and kidney, whereas LXRβ is more ubiquitous (14). The major endogenous LXRs agonists that have been identified are oxysterols [20(S)-, 22(R)-, 24(S)-, 25-, 27-hydroxycholesterol; 24(S), 25-epoxycholesterol] and cholesterol biosynthetic intermediates [desmosterol, follicular fluid meiosis-activating sterol (FF-MAS), or testis meiosis-activating sterol (T-MAS)] (15, 16). Two synthetic LXRα/β agonists were developed and have been used widely such as T0901317 and GW3965 (17). The physiologically most important oxysterols are generated in cells by mitochondrial or endoplasmic reticulum cholesterol hydroxylases belonging to the cytochrome P450 family (CYP46A1, CH25H, CYP27A1 CYP3A4, and CYP7A1). Several natural compounds derived from plants have also been shown to activate (phytosterols, diterpenes…) or inhibit (naringenin) LXRs activity (15). Studies from LXR-knockout mice have demonstrated that LXRs are important in the control of cholesterol homeostasis. Activation of LXRs protects cells from cholesterol accumulation by suppressing cholesterol biosynthesis, activating the conversion of cholesterol to bile acids in the liver, and decreasing intestinal cholesterol absorption (18, 19). Indeed, LXRα-knockout mice fed with a high-cholesterol diet show accumulation of cholesteryl-esters in the liver (20). LXRα is the prevalent isoform acting as hepatic sensor of cholesterol. While *Lxr*α knock-out mice showed an impaired hepatic function when fed high fat diet, mice lacking *Lxr*β are able to resist to the deleterious impact of diet enriched in cholesterol (20, 21).

Cholesterol is the precursor for bile acids (BAs) synthesis within the liver. These molecules are the main constituents of bile and ensure solubilisation and emulsification of fat to help digestion (22). Their synthesis and excretion are major mechanisms of cholesterol catabolism in mammals. Enzymatic modifications of cholesterol result in the production of primary BAs: cholic acid (CA) and chenodeoxycholic (CDCA) (23). There are two main BA synthesis pathways (24). The ≪classical≫ pathway involves CYP7A1 and CYP8B1 enzymes while the ≪alternative≫ pathway involves the cholesterol 27-hydroxylase CYP27A1 and 25-hydroxycholesterol 7-alpha-hydroxylase CYP7B1. Before their excretion, BAs are, in part, conjugated with glycine or taurine in the liver. This leads to the production of tauro-, or glyco-conjugates BAs. Stored in the gallbladder, primary BAs and conjugates are discharged during the meal into the duodenum to facilitate fat digestion. In the ileum, BAs are in part deconjugated and modified to give rise to secondary BAs (25). Deoxycholic acid (DCA) and lithocholic acid (LCA) are derived respectively from CA and CDCA. BAs have been described as signaling molecules that signal through two main receptors: the nuclear Farnesoid X receptor alpha (FXRα; NR1H4) (26–28) and the membrane G protein-coupled bile acid receptor TGR5 (GPBAR1) (24, 29).

In addition, the nuclear receptor FXRα plays important role on cholesterol homeostasis through the regulation of bile acid metabolism in the liver and in the intestine. In the liver, FXR reduces the expression of the genes encoding key enzymes of bile acids biosynthesis pathways: the cholesterol 7α-hydroxylase (Cytochrome P450 7a1; CYP7A1) and the sterol 12α-hydroxylase (CYP8B1). In the intestine, FXRα activates the expression of the Fibroblast Growth Factor 19 (FGF19 in human) or FGF15 (mouse) which acts in an endocrine manner on hepatocytes *via* the Fibroblast Growth Factor Receptor 4 (FGFR4) to repress *Cyp7a1* expression (30, 31).

## The testis, cholesterol homeostasis

The testis produces male gametes, namely spermatozoa. It is composed of a network of seminiferous tubules, separated from each other by the interstitial space. These two compartments define the two major functions of the testis with the production of gametes (exocrine function) and sexual hormone (endocrine function). Leydig cells within the interstitial space provide part of the endocrine function, and the exocrine function takes places within the seminiferous tubules. Their efficiencies are based on the specific actions of the different cell types and their interactions.

The purpose of this special issue is to collect recent acquisition on the role of cholesterol and/or derivatives as signaling molecules, whose alteration can determine the onset or progression of different pathologies. Here, we will highlight the roles of pathways activated by cholesterol and derivatives through nuclear receptors on male fertility disorders.

### The testicular endocrine function

In human and rodents, testosterone is the main type of circulating androgens, which is mainly synthesized by the Leydig cells. Testis also produces small amounts of other hormones such as estrogen and progesterone.

Maintenance of an adequate concentration of intra-testicular testosterone is essential for testicular function, especially for spermatogenesis (32). The hypothalamo-pituitary axis exerts a major control on testicular steroidogenesis. Gonadotropin Releasing Hormone (GnRH) secreted by the hypothalamus, stimulates the synthesis and release of Luteinizing Hormone (LH) and Follicle Stimulating Hormone (FSH) by the gonadotropic cells of the anterior pituitary (33). The LH acts directly on the Leydig cells to control their steroidogenic activity *via* the activation of the luteinizing hormone/choriogonadotropin receptor, LHCGR. In return, sex steroids exert a negative feedback on their own synthesis. LH is involved in the regulation of steroidogenesis at different levels. LH pathway promotes the activation of cholesterol ester hydroxylase increasing the free cholesterol pool (34) and also promotes the transfer of cholesterol from the outer membrane to the inner membrane of the mitochondria. LH signaling pathway leads to the transcriptional activation of LH target genes such as *StAR* (35), *Cyp11a1* (36), and *3*β*Hsd* (37).

The major role of cholesterol in the endocrine function has been well established, as cholesterol is the precursor of steroids. There are multiple sources of cholesterol that contribute to steroidogenesis such as *de novo* synthesized, (b) stored cholesteryl esters, exogenous lipoprotein-supplied cholesterol as well as plasma membrane-derived cholesterol. The plasma lipoproteins are the main source of steroid synthesis. The homeostasis of the interstitial tissue, mainly in Leydig cells, involved factors of cholesterol homeostasis such as HMG-CoA reductase, HSL, and ACAT (38, 39). Among others, the links between cholesterol and steroid synthesis are highlighted by the control of the SCAP/SREBP pathway, a regulator of cholesterol homeostasis by cAMP the secondary messenger of the LH pathways.

Sex steroid hormones share a common biosynthesis pathway, with cholesterol as unique precursor. Leydig cells are able to *de novo* synthesize cholesterol or can also use stored cholesterol ester to ensure steroidogenesis function (40). A recent study shows that the plasma membrane is one of the richest source of free cholesterol available for acute steroidogenesis (41). The first enzymatic step of steroidogenesis involves the conversion of cholesterol to pregnenolone, which occurs within mitochondria. It requires the transport of cholesterol from the outer mitochondrial membrane to the inner membrane. This limiting step is mainly provided by the Steroidogenic Acute Regulatory protein (STAR) (42). This crucial point has been highlighted by the use of transgenic mouse models. Mice lacking *Star* present gonadal insufficiencies due to steroidogenesis defects associated with lipid accumulation in the interstitial compartment (43). Recently, using a mitochondrial reconstitution assay system, it has been shown that SNARE proteins (Soluble N-ethylmaleimide-sensitive-factor Attachment *protein* REceptor) are vital components contributing to cholesterol transport within the mitochondria (44). Then, the biosynthesis involves the oxidative cleavage of the side-chain of cholesterol by cholesterol side-chain cleavage enzyme (cytochrome P450scc, CYP11A1), a mitochondrial cytochrome P450 oxidase, to give pregnenolone (45). Pregnenolone may be converted to testosterone through two pathways called Delta 4 (Δ4) and Delta 5 (Δ5) (46). The Delta 4 (Δ4) pathway involves conversion of pregnenolone to progesterone by the action of the enzyme 3β-Hydroxysteroid Dehydrogenase (3βHSD). Progesterone is then metabolized by cytochrome P450 17α-hydroxylase/17,20 lyase (CYP17A1) in androstenedione, substrate of the 17β-hydroxysteroid dehydrogenase enzyme (17βHSD) for testosterone synthesis. The Delta 5 (Δ5) pathway is initiated by the CYP17A1 enzyme, that allows the conversion of pregnenolone to dehydroepiandrosterone (DHEA). Then, 17βHSD and 3βHSD enzymes ensure its conversion into androstenediol or androstenedione, and testosterone. The 3βHSD enzyme allows switching from Δ5 to Δ4 pathway. In primates including humans, the Δ5 pathway predominates because CYP17A1 enzyme has a low activity 17, 20 lyase for the conversion of 17α-hydroxyprogesterone to androstenedione (47, 48). A recent proteomic analysis of murine testis lipid droplets (LDs) revealed that these LDs contained a large number of enzymes involved in metabolism and steroidogenesis. Testicular LDs could be an active organelle functionally involved in steroidogenesis (49).

Androgens exert autocrine action on Leydig cells via the androgen receptor (AR) to control their own synthesis. The AR knockout mice in Leydig cells have decreased plasma testosterone concentrations, despite high rates of LH. This hypo-androgenicity is explained by a defect in the expression of key genes encoding steroidogenic enzymes *Cyp17a1, 3*β*hsd*, and *17*β*hsd*. This was shown to be associated with an arrest of spermatogenesis at the stage of round spermatid causing infertility (50, 51). In humans, mutations on the gene encoding the receptor of adrogen AR (*NR3C4*) result in androgen resistance syndrome (52). Estrogen action is also important in regulating testicular function. Estrogen mainly mediated their signal through the activation of two specific estrogen receptors (ER), ERα and ERβ (53, 54). Estrogen effects on steroidogenesis have been elucidated using mouse model. It has been shown that ERα regulates fetal and neonatal steroidogenesis and ERβ is implicated in the control of neonatal gametogenesis. *Er*α is express in Leydig cells during fetal and neonatal life and its deletion increases testosterone production during fetal life from 13.5 dpc (55). In human, estrogens also play a crucial role in the control of reproductive functions. Studies in patients with inactivating mutations of *ER*α or *CYP19* (aromatase cytochrome P450) suggest a role of estrogens signaling in human male fertility (56). Next to the steroid receptors, it has been shown that numerous nuclear receptors are implicated in the regulation of steroids synthesis. This was recently well reviewed (57, 58). Nuclear receptor family has been described to locally control the expression and activity of enzymes involved in steroidogenesis. Among these, the Steroidogenic Factor 1 (SF-1) and the Liver Receptor Homolog 1 (LRH-1) are positive regulators of hormone steroids synthesis. They stimulate the expression of common target genes such as *Star, Cyp11a1, Cyp17a1*, and *3*β*hsd* encoding enzymes of steroidogenesis (59–62). SF-1 and LRH-1 are also involved in the regulation of cholesterol biosynthesis by regulating the expression of *Sr-b1* (scavenger receptor class B type I) gene which provides plasma cholesterol to steroidogenic tissues (63, 64), and the enzyme Hydroxymethylglutaryl-CoA synthase and reductase (HMG CoA) involved in the biosynthesis of cholesterol (65, 66).

In addition, it has been shown that LXRα is expressed within Leydig cells (8, 67, 68). It was demonstrated that in 2.5-month-old LRXα-deficient mice, testosterone production is significantly lower than in wild type control mice. This was associated with a decrease of the expression of *3*β*hsd* enzyme (67). Interestingly, LXR agonist T0901317 increased testosterone concentration in wild-type mice in association with an accumulation of StAR at both mRNA and protein levels (67).

Next to this, a negative regulation of steroid synthesis involving receptors of bile acids has been defined as an important regulator of testicular physiology. BAs receptors, FXRα and TGR5, are expressed within the testis (69, 70) and have been recently shown to be implicated in testis dysfunction (71). Detectable levels of bile acids have been measured in the testis of mice in normal physiological conditions (72) and recently, it was shown that testis expresses key enzymes of BAs synthesis and is able to produce BAs (73).

The bile acids nuclear receptor FXRα is implicated in steroidogenesis regulation. In 2007, the presence of FXRα within mouse Leydig cells was demonstrated for the first time (69). While *Fxr*α^−/−^ male presented the same plasma testosterone level than control mice, induction of FXRα with a synthetic agonist in wild type mice results in a decrease of androgen production independently of the hypothalamo-pituitary axis. The activation of the FXRα receptor in Leydig cells induces transcription of the gene encoding the orphan nuclear receptors SHP (Small Heterodimer Partner, *Nr0b2*) and DAX-1 (dosage-sensitive sex reversal, adrenal hypoplasia critical region, on chromosome X, gene 1, *Nr0b1*), two orphan nuclear receptors, which are negative regulators of steroidogenic Leydig cells activity. Indeed, SHP AND DAX-1 inhibit the expression of *Lrh1* and *Sf1*. SHP also interacts with LRH1 thereby inhibiting its trans-activating activity. This decreases expressions of genes encoding key enzymes of steroidogenesis and thus leads to lower testosterone levels (69). In that line, through the regulation of the endocrine function, it has been demonstrated that BA-FXRα pathways also regulate male sexual maturation (74). Mice fed a diet supplemented with BAs during pubertal age show increased incidence of infertility. This is associated with altered differentiation and increase apoptosis of germ cells due to lower testosterone levels. This is sustained by the fact that supplementation with testosterone abolished the effect of CA-diet on germ cell apoptosis.

**Figure 2:**
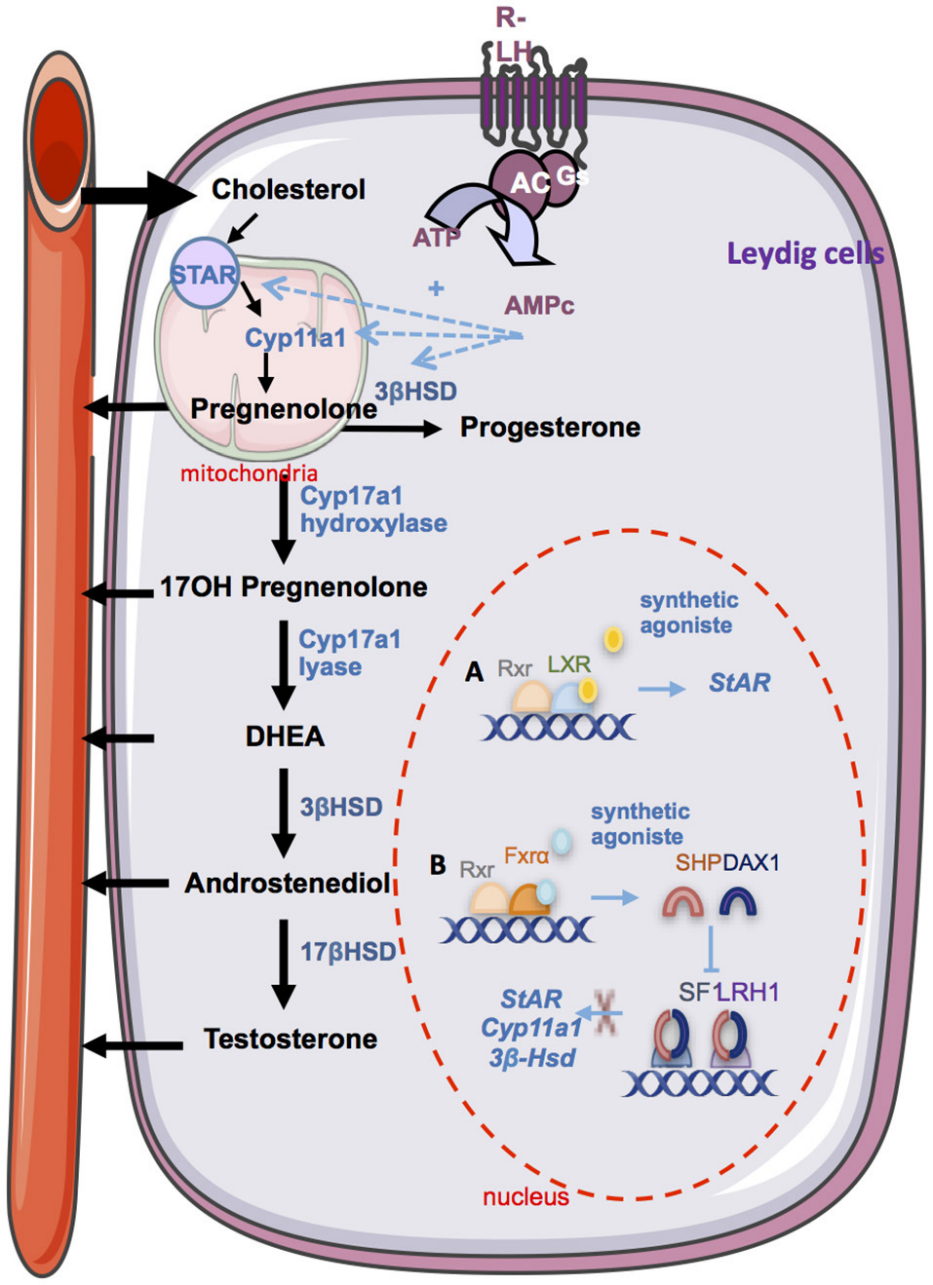
The endocrine function. Steroidogesesis occurs in Leydig cells from cholesterol. The first step involves the STAR protein which allow the transport of cholesterol within the mitochondria. Then, cholesterol is converted in pregnenolone by the CYP11A1 enzyme. Several enzymatic transformation steps will convert pregnenolone into testosterone. The differents enzymes are marked in blue. LH via it receptor (R-LH) induces the increase expression of some key enzymes of spermatogenesis. Some nuclear receptors have been described as regulators of spermatogenesis. **(A)** The activation of LXRs by a synthetic agonist induces the upregulation of Star. **(B)** The activation of FXRα by a synthetic agonist induce the increase *Shp* of *Dax1* which in turn inhibits the trans activating activity of SF1 and LRH1 on the promotor of steroidogenic enzyme such as *Star, Cyp11a1*, and *3bhsd*. STAR, steroidogenic acute regulatory protein; Cyp11a1, cytochrome p450scc cholesterol side chain clivage; Cyp17a1, cytochrome P450 17α-hydroxylase/17,20 lyase; 3βHsd, 3β·hydroxysteroid dehydrogenase; 17βHsd, 17β-hydroxysteroid dehydrogenase; DHEA, dehydroepiandrosterone; RXR, retinoid acid receptor; LXR, liver X receptor; FXR, farnesoid X receptor; Shp, small heterodimer partner; Dax1, dosage-sensitive sex reversal, adrenal hypoplasia critical region, on chromosome X, gene 1; SF1, steroidogenic factor 1; LRH1, liver receptor homolog 1.

These overall data clearly suggest a complex regulation of testicular steroidogenesis by nuclear receptors whose activities are regulated by cholesterol derivatives such as oxysterols and bile acids (Figure 2).

### The testicular exocrine function

**Figure 3:**
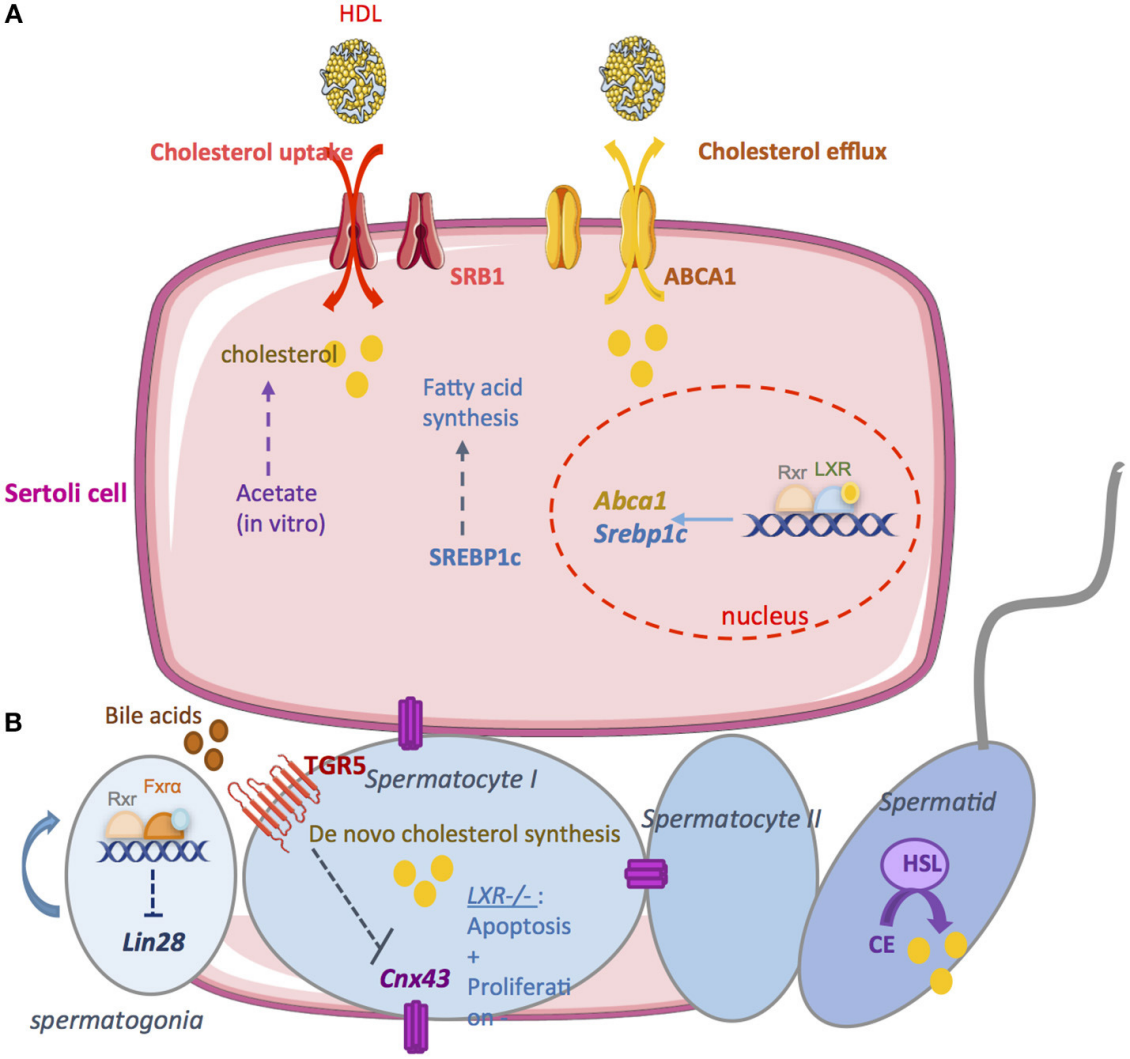
The exocrine function. **(A)** A large amount of lipids used for spermatogenesis are provided by Sertoli cells. *In vitro*, it has been demonstrated that Sertoli cells are able to synthesize cholesterol from acetate. Therefore, it is necessary to import cholesterol from the blood circulation. high density lipoprotein (HDL) is the primary source of cholesterol used by Sertoli cells. SRB1 facilitates the uptake of HDL within mitochondria. Sertoli cells maintain cholesterol homeostasis through reverse cholesterol transport which relies on the cholesterol transporter ATP-binding cassette A1 (ABCA1). LXR/RXR heterodimers increase the cellular cholesterol content by enhancing the levels of SREBP-1c and decrease cholesterol levels by increasing ABCAI expression. **(B)** Germ cells are able to produce cholesterol *de novo*. Cholesterol *de novo* synthesis was increased during the development of pachytene, leptotene, and zygotene stages of spermatocyte I. During the latest steps, the hormone sensitive lipase (HSL) mediates the hydrolysis of cholesterol esters. The bile acids nuclear receptor FXRα regulate germ cell physiology. FXRα inhibits the expression of the pluripotency marker Lin28. FXR^−/−^ mice present an increase number of undifferenciated spermatogonia that induce an increase number of spermatozoa. The G coupled bile acids receptor TGR5 also regulates germ cell physiology. Activation of TGR5 by Bile acids in spermatocytes decrease Cnx43 expression inducing the rupture of blood testis barrier. Nuclear receptor for oxysterols LXRs are also important regulators of germ cells physiology. In LXR^−/−^ mice, germ cells apoptosis increase while there is a decrease in the proliferation rate. This could explain the complete loss of germ cells in these mice leading to infertility.

Germ cell differentiation is closely related to somatic Sertoli cells functions, which provide structural and nutritional supports (Figure 3).

#### The sertoli cells

One primary function of Sertoli cells is to set up the blood testis barrier (BTB) at puberty under the action of androgens, retinoids and thyroid hormones (75–77). The BTB is composed of specialized junctions established between adjacent Sertoli cells near the basement membrane of seminiferous tubules. That defines Sertoli cells polarity giving them different structural and functional poles. The BTB divides the seminiferous tubules in basal and apical compartments. Self-renewal and differentiation of spermatogonia as well as progression to the stage pre-leptotene spermatocyte I occur in the basal compartment. The two meiotic divisions and post-meiotic steps take place in the apical compartment. The BTB regulates and restricts the passage of nutritional substances, vital molecules, and toxic compounds in the apical compartment of the seminiferous tubules. BTB also defines an immune privileged environment. It isolates many antigens present on the surface of post-meiotic germ cells from the systemic circulation thus preventing the emergence of the corresponding antibodies and autoimmune diseases leading to infertility (78). Sertoli cells also participate to the secretion of components of the extracellular matrix necessary to maintain the integrity of the seminiferous epithelium and intercellular junctions (79). In addition, they synthesize proteases and protease inhibitors contributing to the dynamic of junction complexes allowing the migration of germ cells (80). Sertoli cells provide a set of essential nutrients for germ cells survival and differentiation, including amino acids, carbohydrates, lipids and vitamins (80). Sertoli cells are able to metabolize glucose into pyruvate and lactate (81) which is the preferential energy substrate for post-meiotic germ cells to support their metabolic activity. Sertoli cells secrete numerous growth factors modulating the proliferation and/or differentiation of germ cells (82). In addition Sertoli cells secrete severals transport proteins that will ensure the passage of metal ions (Transferrin) (83) or hormone (androgen-binding protein, retinoid binding protein) (84) through the BTB for the metabolic requirement of the post-meiotic germ cells.

Sertoli cells provide large amounts of lipids used for spermatogenesis. *In vitro*, it has been demonstrated that Sertoli cells are able to synthesize cholesterol from acetate (85). However, this *de novo* synthesis is not a sufficient source of cholesterol to ensure spermatogenesis *in vivo*. Therefore, it is necessary to import cholesterol from the blood circulation. In rodent, it has been demonstrated that high density lipoprotein (HDL) is the primary source of cholesterol used by Sertoli cells (86). The high-density lipoprotein transporter SR-B1 is essential to import cholesterol from blood. SR-B1 is an integral membrane glycoprotein expressed in Leydig and Sertoli cells (87, 88). This transporter facilitates the uptake of HDL. It has been demonstrated that the overexpression of SR-B1 is associated with an increase of esterified cholesterol in Sertoli cells (89). SR-B1 is also implicated in the phagocytosis of apoptotic germ cells. This may also increase cholesterol levels in Sertoli cells (90). Another important point is the elimination of cholesterol overflow. Cholesterol is esterified and stored in lipid droplets to limit toxicity. Sertoli cells maintain cholesterol homeostasis through reverse cholesterol transport. This reverse transport primarily relies on the cholesterol transporter ATP-binding cassette A1 (ABCA1) (91). Genetic mutations in *ABCA1* lead to the absence of HDL in the plasma and the accumulation of esterified cholesterol in tissues, increasing the incidence of atherosclerosis (92). ABCA1 is highly expressed in the testis and it was observed that within the seminiferous tubules, ABCA1 is more expressed in Sertoli cells (91). Authors demonstrated that TM4 Sertoli cells lacking *Abca1* or primary Sertoli cells cultured from *Abca1*^−/−^ mice fail to efflux cholesterol. *In vivo, Abca1*^−/−^ mice present lipid accumulation in Sertoli. At 6 months, intra-testicular testosterone levels and sperm counts are significantly reduced in *Abca1*^−/−^ mice compared with controls. Thereby, the fertility of *Abca1*^−/−^ mice is reduced across their reproductive lifespans. These results indicate that ABCA1 play important role to Sertoli cells function and male fertility.

Regarding the cellular signaling, it has been demonstrated that LXRβ is expressed in Sertoli cells. The *Lxr*β^−/−^ knockout mice are fertile with few observed defects (57, 67). However, these mice present cholesteryl ester accumulation in the Sertoli cells. This is associated with a decrease of germ cell proliferation rate. Interestingly, it was shown that men with azoospermia present a significant decrease of *Lxr*β expression within the testis associated with fewer proliferating germ cells (93). Mice deficient for both LXRs isoforms present progressive testicular degeneration associated with germ cell depletion. *Lxr*α^−/−^;*Lxr*β^−/−^ are reported to be infertile by 7–9 months of age (67, 68). As in *Lxr*β^−/−^ mice, lipid accumulation in Sertoli cells appears to be the earliest phenotype of *Lxr*α^−/−^;*Lxr*β^−/−^ mice. A recent study further investigate the roles of LXRβ in Sertoli cells by generating a mouse model that re-expresses *Lxr*β only in Sertoli cells in the *Lxr*α^−/−^;*Lxr*β^−/−^ mice (94). Authors showed that this *Lxr*β re-expression is fundamental for Sertoli cells to maintain functional BTB and to sustain the germ cell pool. As expected, *Lxr*β re-expression restored lipid homeostasis in Sertoli cells and regulates the endocrine function of Leydig cells. However, these mice are infertile with spermatogenesis defects. Furthermore, it was also demonstrated that wild-type animals exposed to the synthetic LXR agonist T0901317, within diet, exhibited increased levels of the LXR target genes sterol regulatory element-binding protein-1c (SREBP-1c) and ABCA1 (14, 95). Another study comforts the previous results in MSC-1 cells treated with T0901317 where ABCA1 levels were also increased (68).

These data clearly demonstrate the main roles of LXRs in the regulation lipid homeostasis of Sertoli cell and thus male fertility.

#### The germ cell lineage

Spermatogenesis is a highly coordinated process leading to the production of male haploid gametes differentiated from diploid germ stem cells. Spermatogenesis begins with a proliferative phase with the differentiation of type A spermatogonia located in contact with the basement membrane of the seminiferous tubules. A_s_ (A single) spermatogonia possess the ability to renew the population of undifferentiated germ cells. Spermatogonia can also enter in the differentiation process. Then spermatogonia engaged in meiosis, the diploid spermatocyte gives rise to haploid cells called secondary spermatocyte or spermatocyte II and then round spermatids. Meiosis step is followed by spermiogenesis; which is the terminal process of spermatogenesis differentiation. The spermatids nucleus will be reorganized leading to chromatin compaction (96). Firstly the majority of histones is replaced by small basic transition proteins (TNP1 and TNP2). Secondly, transition proteins will be replaced by specific testis nuclear protein, protamines (PRM1 and PRM2) (97) to protect the genome from physical and chemical attack.

There is an intimate association between cholesterol metabolism and fertility during spermatogenesis. Cholesterol is required for the mass production of germ cells during spermatogenesis. A study determined that cholesterol *de novo* synthesis was increased during the development of pachytene, leptotene, and zygotene stages (98), which was associated with increased diameter and surface area of germ cells. In later stages, the rate of cholesterol synthesis tends to decrease and remains low throughout the following phases of spermatogenesis. During the last steps of spermatogenesis the Hormone-sensitive lipase (HSL) is known to mediate the hydrolysis of cholesterol esters. It has been shown that the HSL is localized in elongating spermatids and spermatozoa. The most striking phenotype of *Hsl*^−/−^ mice is male sterility caused by oligospermia (99). *Hsl*^−/−^ mice were found to accumulate diacylglycerols and cholesterol esters in the testis. A recent study further investigate *Hsl*^−/−^ mice testis. Authors showed that spermatogenesis is arrested just before spermiogenesis elongation step (100). An important pathway of lipid metabolism must be lost in *Hsl*^−/−^ testes causing an arrest of cell differentiation.

Like any animal cell, spermatozoa have a lipid bilayer plasma membrane. The proportion of the different constituents gives to the spermatic plasma membrane unique properties. The spermatic membrane has been shown to be rich in polyunsaturated fatty acids (PUFAs) important for ensuring the viability and mobility of the spermatozoon (101–103). The high proportion of PUFA also seems to play an important role in the process of membrane fusion between spermatozoon and oocyte. Indeed, PUFAs contribute to flexibility and membrane fluidity (104, 105). The high proportion of PUFA, especially docosahexaenoic acid (DHA) in humans, is important for fertility. Indeed, one study showed a decrease in PUFAs and an increase in saturated fatty acids (SFA) in spermatozoa of asthenozoospermic men compared to sperm of normozoospermic men (106). Another study also found a negative correlation between body mass index and DHA and a positive correlation between DHA and normal sperm parameters (107). Sterols are the second major components of the plasma membrane. Cholesterol is the most abundant sterol found in the spermatic membrane of many species (102). Spermatozoa are enriched in cholesterol within the seminiferous tubules. Indeed, in the spermatocyte stage, the germ cells are able to synthesize *de novo* cholesterol to allow the increase of their membrane surface (98). Sertoli cells also participate in this process by providing to sperm cells *de novo* synthesized cholesterol or derived from the bloodstream (85, 89). More recently, the presence of oxidized cholesterol derivatives has been described in several species including mice (108). These oxysterols could influence the membrane fluidity (109). The spermatic membrane is also composed of complex lipids such as phospholipids (PL) and sphingolipids (77, 85). The lipid composition of spermatozoa is of great importance during capacitation in the female genital tract and also in membrane fusion events during fertilization. After spermiation, the lipid composition of the spermatic membrane is not definitive and will undergo many modifications during the epididymal transit and the capacitation process. Lipids largely influence post-testicular maturation of spermatozoa. During the epididymal transit, the lipid composition of the gamete is strongly reshuffled (110–112). These modifications influence the membrane dynamics in order to confer greater membrane fluidity. These lipid rearrangements are finely regulated. The epididymis produces lipid vesicles allowing protein transfer to spermatozoa. Finally, capacitation is a stage of post-testicular maturation partly regulated by the lipid composition of the spermatic membrane (113). Therefore, regulation of the lipid profile of gametes is essential for maintaining male fertility. It has recently been shown that the lipid composition of spermatozoa varies according to the dietary intake of lipids (114), demonstrating a direct correlation between diet and gamete composition.

Mammalian spermatozoa are specialized and polarized cells, with one specific function which is the fertilization of the female oocyte. This fertilizing capacity is acquired during a multistep process after spermiation called post testicular maturation. Spermatozoa maturation starts during its epididymal transit and ends in the female genital tract. The maturation of spermatozoa remains the major function of the epididymis. Epididymal maturation is essential for the acquisition of fertilizing power but is also essential to ensure normal embryonic development (115). Epididymal maturation consists of morphological, functional, and biochemical changes.

Regarding the signaling pathways involved, the LXR signaling pathways control implicated in the regulation of spermatogenesis (67). Spermatogenesis results from a balance between cell proliferation, cell differentiation, and cell death. Proliferation and apoptosis are respectively altered in *Lxr*β^−/−^ and *Lxr*α^−/−^ mice, whereas these mice did not show any fertility troubles. In the *Lxr*β^−/−^ mice the lower proliferation rate was compensated by a decreased apoptosis rate and in *Lxr*α^−/−^ mice the higher apoptosis level was associated with an increased proliferation rate. The lack of both LXRs led to a dramatic decline in proliferation and increase in apoptosis. This could explain the complete loss of germ cells in these mice and thus infertility. Recently, the implication of LXRs in the physiopathology of defective spermatogenesis in non-obstructive azoospermia (NOA) patients was investigated (93). Indeed, abnormalities observed in NOA patients look like those observed in *Lxr*^−/−^ mice. It has been shown that *Lxr* mRNA levels were decreased in NOA specimens and positively correlated with germ cell number. As expected, authors showed a decreased level of *Idol* (Inducible Degrader of the LDL receptor) and *Srebp1c* (Sterol regulatory element-binding protein 1), which are LXR target genes.

Next to this, a recent study has shown that the G coupled bile acids receptor TGR5 was expressed within the testis (70). It has been shown that BA-TGR5 signaling pathways alter testicular epithelium integrity leading to adult male fertility disorders (72). Indeed, after 4 months of dietary cholic acid (CA) supplementation, 25% of male mice were sterile and in fertile ones a 20% decrease of pups per litter was reported. Male exposed to BAs presented lower spermatozoa in the tail of the epididymis explaining the fertility defect. Interestingly, authors showed that *Tgr5*^−/−^ mice were preserved of the CA deleterious effects on the testis. At the molecular level, BAs exposure induced a repression of cell-cell interactions network through the down-regulation of N-cadherin as well as connexin 43 (CX43) expression. This leads to germ cell sloughing, rupture of the BTB and then spermatids apoptosis. In humans, a reduction in the expression of CX43 was shown to be associated with the degree of spermatogenesis defect (116–118).

Recently, it has been shown that bile acids nuclear receptor FXRα regulates germ cell physiology (73). Authors showed that FXRα plays a role in the establishment and maintenance of the undifferentiated germ cell pool that in turn influences male fertility. *Fxr*α^−/−^ males present an extended fertility with aging. In *Fxr*α^−/−^ males, the maintenance of fertility capacities results from a higher number of undifferentiated germ cells during aging associated with high spermatozoa production. FXRα regulates the expression of several pluripotency factors within the germ cell lineage. *In vitro* approaches show that FXRα controls the expression of the pluripotency marker Lin28 in the germ cells.

### Obesity and male fertility

In the last decades infertility has become a global public health issue affecting 15% of all reproductive couples. It has been estimated that 70 million couples worldwide experience subfertility or infertility (119). Male factors are responsible for ~25% of cases of infertility (2). Currently, the etiology of low semen quality is poorly understood. Many physiological, environmental and genetic factors have been implicated (120). Metabolic syndrome (MetS) is a group of risk factors such as high blood pressure, high blood sugar, unhealthy cholesterol levels, and abdominal fat. The association between MetS and male hypogonadism is well established and reviewed (121). Cross-sectional studies have found that between 20 and 64% of obese men have low total testosterone levels (122). A number of studies also show that low testosterone is associated with insulin resistance and an increased risk for diabetes mellitus and MetS in men. Testosterone supplementation seems to be beneficial on inflammation, muscle mass, lipid profile, sexual function, and improves comorbidities of obesity, MetS, or cardiovascular disease in males (121).

Evidence from human studies indicates that male obesity and components of the diet could play an important role in the deregulation of spermatogenesis, sperm maturation, or fertilizing ability. Obese or overweight men could present a decrease sperm quantity, quality, and motility (123, 124). A study showed differences between obese and lean patients regarding the acrosome reaction (125). Sperm acrosome reaction is impaired in obese men associated with altered circulating levels of estradiol and sperm cholesterol content. Obese men can also present an increase sperm DNA damage or lower embryo implantation rate (122, 126). The majority of studies in humans focuses on the impact of obesity on male fertility and do not take into account the plasma lipid profile. It is reported that 65% of infertile men show hypercholesterolemia and/or triglyceridemia (127). The impact of dyslipidemia on male fertility remains controversial. Even if cholesterol and lipid homeostasis is essential for male fecundity (57, 128), a recent study reported that hypercholesterolemia is not associated with sperm concentration or motility in men (129). It has also been shown that there is no correlation between sperm concentration and serum total cholesterol or triglyceride in human (130).

Some animal studies revealed that high cholesterol diet can impair fertility and lower sperm quality. Rabbit fed a High fat diet (HFD) is an experimental model of metabolic syndrome (MetS) that closely similar to the human syndrome (131–133). This model has been characterized by hyperglycaemia, hypercholesterolemia, hypertension, hypogonadotropic hypogonadism, penile alterations (131, 132, 134) or nonalcoholic steatohepatitis. This model is very useful to study the deleterious impact of MetS symptoms such as hypercholesterolemia on male fertility. It has been demonstrated that supplementation with testosterone (131, 132) with the FXRα agonist the obeticholic acid (133, 134) normalize several MetS symptoms including HFD-induced penile alterations. These animals presented abnormal sperm morphology, decreased sperm number and declined percentage of motile sperm or acrosome reaction (134–136, 138, 139). It has been shown that administration of tamoxifen, used in the treatment of idiopathic male infertility to HFD rabbits partially restored sperm motility, but further decreased morphology and increased spontaneous acrosome reaction (136). It has also been shown that hyper-cholesterolemic rabbits present lower testicular efficiency related to both a decrease in spermatogonial cells and an increase in germ cell apoptosis (137). On the other hand, spermiogenesis, which is the last step of spermatogenesis, was also affected in these animals. In post-meiotic germ cells from hyper-cholesterolemic rabbits, abnormal development of acrosome, nucleus, and inaccurate tail implantation were associated with actin–alpha-tubulin–GM1 sphingolipid altered distribution. It was recently shown in rabbits that a cholesterol-enriched diet increased lipid deposition in the seminiferous tubules and disrupted the BTB (138). Total protein levels of the tight-junction protein 1 (ZO-1) and occludin and their distribution patterns were markedly affected, impairing fertility.

A similar study has demonstrated a disruption of BTB in mice exposed to High Fat Diet (HFD) (139). It has been reported that male rodents exposed to HFD present a decreased number of motile spermatozoa associated with a decline of fertilization rates (140–142). Intracellular reactive oxygen species (ROS) and sperm DNA damage were elevated in the HFD group compared with controls that could explain the decrease sperm motility (141). It has been demonstrated that HFD induces a decrease in testosterone production associated with a decrease level of steroidogenic enzyme (143, 144). A recent study suggests that the increase of autophagy process could be a cause of fertility defects induced by HFD (145). Authors demonstrate that inhibiting autophagy with chloroquine improves the decreased fertility of HFD male mice. Furthermore, the excessive activation of autophagy was also observed in sperm samples from obese, sub-fertile male patients.

In addition, some studies were interested in the impact of paternal or maternal exposure to HFD on male offspring (146, 147). It has been shown that preconception exposure of fathers to HFD impairs the motility of spermatozoa of male offspring, despite their control diet consumption (146). Feeding females with a HFD through pregnancy and lactation induces a decrease on LH and testosterone levels in male offspring (147). If translatable to human health, these studies suggest that reproductive defects may be amplified throughout generations because of calorie dense diet.

Interestingly, in the last decade, it has been proposed that derivatives of bile acids (BAs) could be interesting molecules for the treatment of metabolic diseases such as diabetes or obesity. Regarding the links between BA signaling pathways and male testicular physiology (72–74), a study has investigated the consequences of a long term exposure to molecules that activate BA signaling pathways to treat obesity. For that purpose, mice were fed HFD to induce obesity and concomitantly treat with BAs (148). Even if BAs improves abnormalities induced by the HFD such as body weight, glycemia, or lipidic profiles, co-exposure of HFD and BAs leads to a higher level of infertility. This was associated with altered germ cell proliferation, default of endocrine function and abnormalities in cell-cell interactions within the seminiferous tubule.

## Conclusions

Male fertility disorders represent serious health problem and in 30% of the cases the cause remains unknown. Understanding the various pathologic mechanisms causing male infertility represents an active area of research. As reviewed here, cholesterol homeostasis is crucial for testicular functions such as steroidogenesis, Sertoli cells function, or germ cell differentiation. Thus, altered concentrations of plasma cholesterol can affect the reproductive function leading to male infertility. This link between lipid homeostasis and male fertility disorders is clearly evident in patients suffering from hyperlipidemia or metabolic syndrome. Lipid-lowering drugs could probably ameliorate some reproductive symptoms. However, epidemiological data are currently scarce and controversial.

All these data could help in proposing new molecular markers to diagnose cases of infertility or to define whether the results obtained on the incriminated nuclear receptors LXRs and FXRα which can be modulated by ligands can lead to concepts transferable to the clinic either to develop new strategies to treat infertile men or new approached for male contraception. Such perspectives will require the development of testicular-specific LXR or FXR modulators to treat testicular disorders and therefore male disorders without causing their impacts that may lead to metabolic disorders.

In the opposite way, modulating some of these receptors through the use of agonist or antagonist molecules would improve the symptoms of metabolic syndrome diseases. However, it has been shown in mice that modulating nuclear receptors signaling induce deleterious effects on testicular physiology leading to infertility. In that line, as molecules modulating the BA receptor signaling pathways have been proposed to treat metabolic diseases, it will be important to define the molecular mechanisms involved in the deleterious impact of BAs on testis physiology. Such studies will be essential to define how to pharmacologically or genetically modulate bile acid receptors to treat metabolic disorders while minimizing impacts on male reproductive functions. In addition, the identification of the crosstalk between increased BA levels and metabolic diseases on fertility capacities of male mice should be a mechanism explaining rare clinical situations of infertility following bariatric chirurgical approach in some obese men.

It will be interesting in the future to identify at the clinical levels, using biopsies of patients suffering from liver disorders (cholestasis, hepatitis…), the potential involvement of bile acid signaling pathways in the human regulation of male fertility. According to previous published data, it will be necessary to analyse both BA levels as well as the BA pool composition. Indeed, it appears that secondary BA, namely DCA, is the one whose levels are inversely correlated with fertility. Such analyses on human cohort will be useful to define if BA levels or pool composition might be a useful biomarker linking liver disorders and male infertility; or even if BA could be markers of idiopathic infertility.

In the recent decades, assisted medical procreation (AMP) has been importantly developed to overpass the fertility disorders. Next to *In vitro* fecundation, important efforts have been done in order to develop approach of *in vitro* production of germ cells from spermatogonial stem cells. This strategy is important to propose AMP to azoospermic men. The recent data regarding the role of FXRalpha in the establishment of the pool of undifferentiated spermatogonia, suggest that FXRalpha modulators might be useful in such approaches to increase the rate and efficiency to produce male games.

## Author contributions

LS, LT, SM, MG, FC, JML, CB and DV have participated to the writing of this review.

### Conflict of interest statement

The authors declare that the research was conducted in the absence of any commercial or financial relationships that could be construed as a potential conflict of interest.

## References

[1] Agarwal A Mulgund A Hamada A Chyatte MR. A unique view on male infertility around the globe. Reprod Biol Endocrinol. (2015) 13:37. 10.1186/s12958-015-0032-1 25928197 PMC4424520

[2] Sharlip ID Jarow JP Belker AM Lipshultz LI Sigman M Thomas AJ. Best practice policies for male infertility. Fertil Steril. (2002) 77:873–82. 12009338 10.1016/s0015-0282(02)03105-9

[3] Jarvi K Lo K Grober E Mak V Fischer A Grantmyre J. The workup and management of azoospermic males. Can Urol Assoc J. (2015) 9:229–35. 10.5489/cuaj.3209 26316904 PMC4537331

[4] Cavallini G. Male idiopathic oligoasthenoteratozoospermia. Asian J Androl. (2006) 8:143–57. 10.1111/j.1745-7262.2006.00123.x 16491265

[5] Parton RG Hancock JF. Lipid rafts and plasma membrane microorganization: insights from Ras. Trends Cell Biol. (2004) 14:141–7. 10.1016/j.tcb.2004.02.001 15003623

[6] Simons K Ikonen E. How cells handle cholesterol. Science (2000) 290:1721–6. 11099405 10.1126/science.290.5497.1721

[7] Yokoyama S. Release of cellular cholesterol: molecular mechanism for cholesterol homeostasis in cells and in the body. Biochim Biophys Acta. (2000) 1529:231–44. 10.1016/S1388-1981(00)00152-9 11111092

[8] El-Hajjaji FZ Oumeddour A Pommier AJC Ouvrier A Viennois E Dufour J. Liver X receptors, lipids and their reproductive secrets in the male. Biochim Biophys Acta (2011) 1812:974–81. 10.1016/j.bbadis.2011.02.004 21334438

[9] Wechsler A Brafman A Shafir M Heverin M Gottlieb H Damari G. Generation of viable cholesterol-free mice. Science (2003) 302:2087. 10.1126/science.1090776 14684813

[10] Goldstein JL Brown MS. Regulation of the mevalonate pathway. Nature (1990) 343:425–30. 10.1038/343425a0 1967820

[11] Goldstein JL DeBose-Boyd RA Brown MS. Protein sensors for membrane sterols. Cell (2006) 124:35–46. 10.1016/j.cell.2005.12.022 16413480

[12] Goedeke L Fernández-Hernando C. Regulation of cholesterol homeostasis. Cell Mol Life Sci. (2012) 69:915–30. 10.1007/s00018-011-0857-5 22009455 PMC11114919

[13] Calkin AC Tontonoz P. Transcriptional integration of metabolism by the nuclear sterol-activated receptors LXR and FXR. Nat Rev Mol Cell Biol. (2012) 13:213–24. 10.1038/nrm3312 22414897 PMC3597092

[14] Repa JJ Liang G Ou J Bashmakov Y Lobaccaro JM Shimomura I. Regulation of mouse sterol regulatory element-binding protein-1c gene (SREBP-1c) by oxysterol receptors, LXRalpha and LXRbeta. Genes Dev. (2000) 14:2819–30. 11090130 10.1101/gad.844900 PMC317055

[15] Komati R Spadoni D Zheng S Sridhar J Riley KE Wang G. Ligands of therapeutic utility for the liver X receptors. Molecules (2017) 22:e88. 10.3390/molecules22010088 28067791 PMC5373669

[16] Mutemberezi V Guillemot-Legris O Muccioli GG. Oxysterols: from cholesterol metabolites to key mediators. Prog Lipid Res. (2016) 64:152–69. 10.1016/j.plipres.2016.09.002 27687912

[17] Ratni H Wright MB. Recent progress in liver X receptor-selective modulators. Curr Opin Drug Discov Devel. (2010) 13:403–13. 20597026

[18] Wang Y Rogers PM Su C Varga G Stayrook KR Burris TP. Regulation of cholesterologenesis by the oxysterol receptor, LXRalpha. J Biol Chem. (2008) 283:26332–9. 10.1074/jbc.M804808200 18676367 PMC2546536

[19] Baranowski M. Biological role of liver X receptors. J Physiol Pharmacol. (2008) 59(Suppl. 7):31–55. 19258656

[20] Peet DJ Turley SD Ma W Janowski BA Lobaccaro JM Hammer RE. Cholesterol and bile acid metabolism are impaired in mice lacking the nuclear oxysterol receptor LXR alpha. Cell (1998) 93:693–704. 9630215 10.1016/s0092-8674(00)81432-4

[21] Alberti S Schuster G Parini P Feltkamp D Diczfalusy U Rudling M. Hepatic cholesterol metabolism and resistance to dietary cholesterol in LXRbeta-deficient mice. J Clin Invest. (2001) 107:565–73. 10.1172/JCI9794 11238557 PMC199420

[22] Poupon R Chignard N Rosmorduc O Barbu V Housset C. [Biliary function and its regulation]. Med Sci. (2004) 20:1096–9. 10.1051/medsci/200420121096 15581462

[23] Russell DW. The enzymes, regulation, and genetics of bile acid synthesis. Annu Rev Biochem. (2003) 72:137–74. 10.1146/annurev.biochem.72.121801.161712 12543708

[24] Martinot E Sèdes L Baptissart M Lobaccaro J-M Caira F Beaudoin C. Bile acids and their receptors. Mol Aspects Med. (2017) 56:2–9. 10.1016/j.mam.2017.01.006 28153453

[25] Ridlon JM Kang D-J Hylemon PB. Bile salt biotransformations by human intestinal bacteria. J Lipid Res. (2006) 47:241–59. 10.1194/jlr.R500013-JLR200 16299351

[26] Makishima M Okamoto AY Repa JJ Tu H Learned RM Luk A. Identification of a nuclear receptor for bile acids. Science (1999) 284:1362–5. 10334992 10.1126/science.284.5418.1362

[27] Parks DJ Blanchard SG Bledsoe RK Chandra G Consler TG Kliewer SA. Bile acids: natural ligands for an orphan nuclear receptor. Science (1999) 284:1365–8. 10334993 10.1126/science.284.5418.1365

[28] Wang H Chen J Hollister K Sowers LC Forman BM. Endogenous bile acids are ligands for the nuclear receptor FXR/BAR. Mol Cell (1999) 3:543–53. 10360171 10.1016/s1097-2765(00)80348-2

[29] Maruyama T Tanaka K Suzuki J Miyoshi H Harada N Nakamura T. Targeted disruption of G protein-coupled bile acid receptor 1 (Gpbar1/M-Bar) in mice. J Endocrinol. (2006) 191:197–205. 10.1677/joe.1.06546 17065403

[30] Inagaki T Choi M Moschetta A Peng L Cummins CL McDonald JG. Fibroblast growth factor 15 functions as an enterohepatic signal to regulate bile acid homeostasis. Cell Metab. (2005) 2:217–25. 10.1016/j.cmet.2005.09.001 16213224

[31] Holt JA Luo G Billin AN Bisi J McNeill YY Kozarsky KF. Definition of a novel growth factor-dependent signal cascade for the suppression of bile acid biosynthesis. Genes Dev. (2003) 17:1581–91. 10.1101/gad.1083503 12815072 PMC196131

[32] Woolveridge I Bryden AA Taylor MF George NJ Wu FC Morris ID. Apoptosis and expression of apoptotic regulators in the human testis following short- and long-term anti-androgen treatment. Mol Hum Reprod. (1998) 4:701–7. 9701793 10.1093/molehr/4.7.701

[33] Shacham S Harris D Ben-Shlomo H Cohen I Bonfil D Przedecki F. Mechanism of GnRH receptor signaling on gonadotropin release and gene expression in pituitary gonadotrophs. Vitam Horm. (2001) 63:63–90. 11358118 10.1016/s0083-6729(01)63003-6

[34] Stocco DM Wang X Jo Y Manna PR. Multiple signaling pathways regulating steroidogenesis and steroidogenic acute regulatory protein expression: more complicated than we thought. Mol Endocrinol. (2005) 19:2647–59. 10.1210/me.2004-0532 15831519

[35] Manna PR Dyson MT Eubank DW Clark BJ Lalli E Sassone-Corsi P. Regulation of steroidogenesis and the steroidogenic acute regulatory protein by a member of the cAMP response-element binding protein family. Mol Endocrinol. (2002) 16:184–99. 10.1210/mend.16.1.0759 11773448

[36] Payne AH. Hormonal regulation of cytochrome P450 enzymes, cholesterol side-chain cleavage and 17 alpha-hydroxylase/C17-20 lyase in Leydig cells. Biol Reprod. (1990) 42:399–404. 2160293 10.1095/biolreprod42.3.399

[37] Keeney DS Mason JI. Expression of testicular 3 beta-hydroxysteroid dehydrogenase/delta 5−4-isomerase: regulation by luteinizing hormone and forskolin in Leydig cells of adult rats. Endocrinology (1992) 130:2007–15. 10.1210/endo.130.4.1312436 1312436

[38] Kabbaj O Holm C Vitale ML Pelletier RM. Expression, activity, and subcellular localization of testicular hormone-sensitive lipase during postnatal development in the guinea pig. Biol Reprod. (2001) 65:601–12. 10.1095/biolreprod65.2.601 11466232

[39] Kabbaj O Yoon SR Holm C Rose J Vitale ML Pelletier R-M. Relationship of the hormone-sensitive lipase-mediated modulation of cholesterol metabolism in individual compartments of the testis to serum pituitary hormone and testosterone concentrations in a seasonal breeder, the mink (Mustela vison). Biol Reprod. (2003) 68:722–34. 12604619 10.1095/biolreprod.102.008169

[40] Shen WJ Azhar S Kraemer FB. Lipid droplets and steroidogenic cells. Exp Cell Res. (2016) 340:209–14. 10.1016/j.yexcr.2015.11.024 26639173 PMC4744538

[41] Venugopal S Martinez-Arguelles DB Chebbi S Hullin-Matsuda F Kobayashi T Papadopoulos V. Plasma membrane origin of the steroidogenic pool of cholesterol used in hormone-induced acute steroid formation in leydig cells. J Biol Chem. (2016) 291:26109–25. 10.1074/jbc.M116.740928 27815506 PMC5207080

[42] Stocco DM. The role of the StAR protein in steroidogenesis: challenges for the future. J Endocrinol. (2000) 164:247–53. 10694364 10.1677/joe.0.1640247

[43] Caron KM Soo SC Wetsel WC Stocco DM Clark BJ Parker KL. Targeted disruption of the mouse gene encoding steroidogenic acute regulatory protein provides insights into congenital lipoid adrenal hyperplasia. Proc Natl Acad Sci USA. (1997) 94:11540–5. 9326645 10.1073/pnas.94.21.11540 PMC23530

[44] Lin Y Hou X Shen WJ Hanssen R Khor VK Cortez Y. SNARE-mediated cholesterol movement to mitochondria supports steroidogenesis in rodent cells. Mol Endocrinol. (2016) 30:234–47. 10.1210/me.2015-1281 26771535 PMC4792230

[45] Hu MC Hsu HJ Guo IC Chung BC. Function of Cyp11a1 in animal models. Mol Cell Endocrinol. (2004) 215:95–100. 10.1016/j.mce.2003.11.024 15026180

[46] Conley AJ Bird IM. The role of cytochrome P450 17 alpha-hydroxylase and 3 beta-hydroxysteroid dehydrogenase in the integration of gonadal and adrenal steroidogenesis via the delta 5 and delta 4 pathways of steroidogenesis in mammals. Biol Reprod. (1997) 56:789–99. 9096858 10.1095/biolreprod56.4.789

[47] Hammar M Petersson F. Testosterone production *in vitro* in human testicular tissue. Andrologia (1986) 18:196–200. 2940941 10.1111/j.1439-0272.1986.tb01761.x

[48] Rey R Campo S Ayuso S Nagle C Chemes H. Testicular steroidogenesis in the Cebus monkey throughout postnatal development. Biol Reprod. (1995) 52:997–1002. 7626726 10.1095/biolreprod52.5.997

[49] Wang W Wei S Li L Su X Du C Li F. Proteomic analysis of murine testes lipid droplets. Sci Rep. (2015) 5:12070. 10.1038/srep12070 26159641 PMC4498221

[50] Tsai EC Matsumoto AM Fujimoto WY Boyko EJ. Association of bioavailable, free, and total testosterone with insulin resistance: influence of sex hormone-binding globulin and body fat. Diabetes Care (2004) 27:861–8. 15047639 10.2337/diacare.27.4.861

[51] Xu Q Lin HY Yeh SD Yu IC Wang RS Chen YT. Infertility with defective spermatogenesis and steroidogenesis in male mice lacking androgen receptor in Leydig cells. Endocrine (2007) 32:96–106. 10.1007/s12020-007-9015-0 17955388

[52] Oakes MB Eyvazzadeh AD Quint E Smith YR. Complete androgen insensitivity syndrome–a review. J Pediatr Adolesc Gynecol. (2008) 21:305–10. 10.1016/j.jpag.2007.09.006 19064222

[53] Green S Walter P Greene G Krust A Goffin C Jensen E. Cloning of the human oestrogen receptor cDNA. J Steroid Biochem. (1986) 24:77–83. 2422449 10.1016/0022-4731(86)90035-x

[54] Koike S Sakai M Muramatsu M. Molecular cloning and characterization of rat estrogen receptor cDNA. Nucleic Acids Res. (1987) 15:2499–513. 3031601 10.1093/nar/15.6.2499 PMC340665

[55] Delbès G Levacher C Duquenne C Racine C Pakarinen P Habert R. Endogenous estrogens inhibit mouse fetal Leydig cell development via estrogen receptor alpha. Endocrinology (2005) 146:2454–61. 10.1210/en.2004-1540 15661855

[56] Carreau S Hess RA. Oestrogens and spermatogenesis. Philos Trans R Soc Lond B Biol Sci. (2010) 365:1517–35. 10.1098/rstb.2009.0235 20403867 PMC2871919

[57] Maqdasy S Baptissart M Vega A Baron S Lobaccaro JMA Volle DH. Cholesterol and male fertility: what about orphans and adopted? Mol Cell Endocrinol. (2013) 368:30–46. 10.1016/j.mce.2012.06.011 22766106

[58] Maqdasy S Trousson A Tauveron I Volle DH Baron S Lobaccaro JMA. Once and for all, LXRα and LXRβ are gatekeepers of the endocrine system. Mol Aspects Med. (2016) 49:31–46. 10.1016/j.mam.2016.04.001 27091047

[59] Chau YM Crawford PA Woodson KG Polish JA Olson LM Sadovsky Y. Role of steroidogenic-factor 1 in basal and 3′,5′-cyclic adenosine monophosphate-mediated regulation of cytochrome P450 side-chain cleavage enzyme in the mouse. Biol Reprod. (1997) 57:765–71. 9314578 10.1095/biolreprod57.4.765

[60] Leers-Sucheta S Morohashi K Mason JI Melner MH. Synergistic activation of the human type II 3beta-hydroxysteroid dehydrogenase/delta5-delta4 isomerase promoter by the transcription factor steroidogenic factor-1/adrenal 4-binding protein and phorbol ester. J Biol Chem. (1997) 272:7960–7. 9065466 10.1074/jbc.272.12.7960

[61] Wang ZN Bassett M Rainey WE. Liver receptor homologue-1 is expressed in the adrenal and can regulate transcription of 11 beta-hydroxylase. J Mol Endocrinol. (2001) 27:255–8. 11564608 10.1677/jme.0.0270255

[62] Sirianni R Seely JB Attia G Stocco DM Carr BR Pezzi V. Liver receptor homologue-1 is expressed in human steroidogenic tissues and activates transcription of genes encoding steroidogenic enzymes. J Endocrinol. (2002) 174:R13–7. 12208674 10.1677/joe.0.174r013

[63] Cao G Zhao L Stangl H Hasegawa T Richardson JA Parker KL. Developmental and hormonal regulation of murine scavenger receptor, class B, type 1. Mol Endocrinol. (1999) 13:1460–73. 10.1210/mend.13.9.0346 10478838

[64] Schoonjans K Annicotte JS Huby T Botrugno OA Fayard E Ueda Y. Liver receptor homolog 1 controls the expression of the scavenger receptor class B type I. EMBO Rep. (2002) 3:1181–7. 10.1093/embo-reports/kvf238 12446566 PMC1308324

[65] Mascaró C Ortiz JA Ramos MM Haro D Hegardt FG. Sterol regulatory element binding protein-mediated effect of fluvastatin on cytosolic 3-hydroxy-3-methylglutaryl-coenzyme A synthase transcription. Arch Biochem Biophys. (2000) 374:286–92. 10.1006/abbi.1999.1600 10666309

[66] Datta S Wang L Moore DD Osborne TF. Regulation of 3-hydroxy-3-methylglutaryl coenzyme A reductase promoter by nuclear receptors liver receptor homologue-1 and small heterodimer partner: a mechanism for differential regulation of cholesterol synthesis and uptake. J Biol Chem. (2006) 281:807–12. 10.1074/jbc.M511050200 16282330

[67] Volle DH Mouzat K Duggavathi R Siddeek B Déchelotte P Sion B. Multiple roles of the nuclear receptors for oxysterols liver X receptor to maintain male fertility. Mol Endocrinol. (2007) 21:1014–27. 10.1210/me.2006-0277 17341595

[68] Robertson KM Schuster GU Steffensen KR Hovatta O Meaney S Hultenby K. The liver X receptor-β is essential for maintaining cholesterol homeostasis in the testis. Endocrinology (2005) 146:2519–30. 10.1210/en.2004-1413 15761042

[69] Volle DH Duggavathi R Magnier BC Houten SM Cummins CL Lobaccaro JMA. The small heterodimer partner is a gonadal gatekeeper of sexual maturation in male mice. Genes Dev. (2007) 21:303–15. 10.1101/gad.409307 17289919 PMC1785120

[70] Vassileva G Golovko A Markowitz L Abbondanzo SJ Zeng M Yang S. Targeted deletion of Gpbar1 protects mice from cholesterol gallstone formation. Biochem J. (2006) 398:423–30. 10.1042/BJ20060537 16724960 PMC1559456

[71] Sèdes L Martinot E Baptissart M Baron S Caira F Beaudoin C. Bile acids and male fertility: from mouse to human? Mol Aspects Med. (2017) 56:101–9. 10.1016/j.mam.2017.05.004 28511935

[72] Baptissart M Vega A Martinot E Pommier AJ Houten SM Marceau G. Bile acids alter male fertility through G-protein-coupled bile acid receptor 1 signaling pathways in mice. Hepatology (2014) 60:1054–65. 10.1002/hep.27204 24798773

[73] Martinot E Sèdes L Baptissart M Holota H Rouaisnel B Damon-Soubeyrand C. The bile acid nuclear receptor FXRα is a critical regulator of mouse germ cell fate. Stem Cell Rep. (2017) 9:315–28. 10.1016/j.stemcr.2017.05.036 28669602 PMC5511114

[74] Baptissart M Martinot E Vega A Sédes L Rouaisnel B de Haze A. Bile acid-FXRα pathways regulate male sexual maturation in mice. Oncotarget (2016) 7:19468–82. 10.18632/oncotarget.7153 26848619 PMC4991395

[75] De França LR Hess RA Cooke PS Russell LD. Neonatal hypothyroidism causes delayed Sertoli cell maturation in rats treated with propylthiouracil: evidence that the Sertoli cell controls testis growth. Anat Rec. (1995) 242:57–69. 10.1002/ar.1092420108 7604982

[76] Tarulli GA Stanton PG Meachem SJ Is the adult Sertoli cell terminally differentiated? Biol Reprod. (2012) 87:13, 1–11. 10.1095/biolreprod.111.095091 22492971

[77] Willems A Batlouni SR Esnal A Swinnen JV Saunders PTK Sharpe RM. Selective ablation of the androgen receptor in mouse sertoli cells affects sertoli cell maturation, barrier formation and cytoskeletal development. PLoS ONE (2010) 5:e14168. 10.1371/journal.pone.0014168 21152390 PMC2994754

[78] Francavilla F Santucci R Barbonetti A Francavilla S. Naturally-occurring antisperm antibodies in men: interference with fertility and clinical implications. An update. Front Biosci. (2007) 12:2890–2911. 17485267 10.2741/2280

[79] Skinner MK Tung PS Fritz IB. Cooperativity between Sertoli cells and testicular peritubular cells in the production and deposition of extracellular matrix components. J Cell Biol. (1985) 100:1941–7. 3889013 10.1083/jcb.100.6.1941 PMC2113598

[80] Mruk DD Cheng CY. Sertoli-Sertoli and Sertoli-germ cell interactions and their significance in germ cell movement in the seminiferous epithelium during spermatogenesis. Endocr Rev. (2004) 25:747–806. 10.1210/er.2003-0022 15466940

[81] Robinson R Fritz IB. Metabolism of glucose by Sertoli cells in culture. Biol Reprod. (1981) 24:1032–41. 6268203 10.1095/biolreprod24.5.1032

[82] Chen SR Liu YX. Regulation of spermatogonial stem cell self-renewal and spermatocyte meiosis by Sertoli cell signaling. Reproduction (2015) 149:R159–67. 10.1530/REP-14-0481 25504872

[83] Sylvester SR Griswold MD. The testicular iron shuttle: a “nurse” function of the Sertoli cells. J Androl. (1994) 15:381–5. 7860417

[84] Porter SB Ong DE Chytil F Orgebin-Crist MC. Localization of cellular retinol-binding protein and cellular retinoic acid-binding protein in the rat testis and epididymis. J Androl. (1985) 6:197–212. 2987169 10.1002/j.1939-4640.1985.tb00836.x

[85] Wiebe JP Tilbe KS. *De novo* synthesis of steroids (from acetate) by isolated rat Sertoli cells. Biochem Biophys Res Commun. (1979) 89:1107–13. 496940 10.1016/0006-291x(79)92122-3

[86] Fofana M Travert C Carreau S Le Goff D. Evaluation of cholesteryl ester transfer in the seminiferous tubule cells of immature rats *in vivo* and *in vitro*. J Reprod Fertil. (2000) 118:79–83. 10793628 10.1530/jrf.0.1180079

[87] Rigotti A Miettinen HE Krieger M. The role of the high-density lipoprotein receptor SR-BI in the lipid metabolism of endocrine and other tissues. Endocr Rev. (2003) 24:357–87. 10.1210/er.2001-0037 12788804

[88] Landschulz KT Pathak RK Rigotti A Krieger M Hobbs HH. Regulation of scavenger receptor, class B, type I, a high density lipoprotein receptor, in liver and steroidogenic tissues of the rat. J Clin Invest. (1996) 98:984–95. 10.1172/JCI118883 8770871 PMC507514

[89] Akpovi CD Yoon SR Vitale ML Pelletier RM The predominance of one of the SR-BI isoforms is associated with increased esterified cholesterol levels not apoptosis in mink testis. J Lipid Res. (2006) 47:2233–47. 10.1194/jlr.M600162-JLR200 16861621

[90] Nakanishi Y Shiratsuchi A. [Physiological role and mechanism of phagocytic clearance of apoptotic cells]. Seikagaku (2003) 75:1429–37. 14699843

[91] Selva DM Hirsch-Reinshagen V Burgess B Zhou S Chan J McIsaac S. The ATP-binding cassette transporter 1 mediates lipid efflux from Sertoli cells and influences male fertility. J Lipid Res. (2004) 45:1040–50. 10.1194/jlr.M400007-JLR200 15026428

[92] Singaraja RR Brunham LR Visscher H Kastelein JJP Hayden MR. Efflux and atherosclerosis: the clinical and biochemical impact of variations in the ABCA1 gene. Arterioscler Thromb Vasc Biol. (2003) 23:1322–32. 10.1161/01.ATV.0000078520.89539.77 12763760

[93] Rondanino C Ouchchane L Chauffour C Marceau G Déchelotte P Sion B. Levels of liver X receptors in testicular biopsies of patients with azoospermia. Fertil Steril. (2014) 102:361–71.e5. 10.1016/j.fertnstert.2014.04.033 24842676

[94] Maqdasy S El Hajjaji FZ Baptissart M Viennois E Oumeddour A Brugnon F. Identification of the functions of liver X receptor-β in sertoli cells using a targeted expression-rescue model. Endocrinology (2015) 156:4545–57. 10.1210/en.2015-1382 26402841

[95] Kennedy MA Venkateswaran A Tarr PT Xenarios I Kudoh J Shimizu N. Characterization of the human ABCG1 gene: liver X receptor activates an internal promoter that produces a novel transcript encoding an alternative form of the protein. J Biol Chem. (2001) 276:39438–47. 10.1074/jbc.M105863200 11500512

[96] Dadoune JP. Expression of mammalian spermatozoal nucleoproteins. Microsc Res Tech. (2003) 61:56–75. 10.1002/jemt.10317 12672123

[97] Hecht NB Regulation of “haploid expressed genes” in male germ cells. J Reprod Fertil. (1990) 88:679–93. 2182846 10.1530/jrf.0.0880679

[98] Potter JE Millette CF James MJ Kandutsch AA. Elevated cholesterol and dolichol synthesis in mouse pachytene spermatocytes. J Biol Chem. (1981) 256:7150–4. 7251590

[99] Osuga J Ishibashi S Oka T Yagyu H Tozawa R Fujimoto A Targeted disruption of hormone-sensitive lipase results in male sterility and adipocyte hypertrophy, but not in obesity. Proc Natl Acad Sci USA. (2000) 97:787–92. 10639158 10.1073/pnas.97.2.787 PMC15409

[100] Wang F Chen Z Ren X Tian Y Wang F Liu C. Hormone-sensitive lipase deficiency alters gene expression and cholesterol content of mouse testis. Reproduction (2017) 153:175–85. 10.1530/REP-16-0484 27920259 PMC5148802

[101] Lenzi A Picardo M Gandini L Dondero F. Lipids of the sperm plasma membrane: from polyunsaturated fatty acids considered as markers of sperm function to possible scavenger therapy. Hum Reprod Update (1996) 2:246–56. 9079417 10.1093/humupd/2.3.246

[102] Ladha S. Lipid heterogeneity and membrane fluidity in a highly polarized cell, the mammalian spermatozoon. J Membr Biol. (1998) 165:1–10. 9705977 10.1007/s002329900415

[103] Neill AR Masters CJ. Metabolism of fatty acids by ovine spermatozoa. J Reprod Fertil. (1973) 34:279–87. 4354967 10.1530/jrf.0.0340279

[104] Fleming AD Yanagimachi R. Evidence suggesting the importance of fatty acids and the fatty acid moieties of sperm membrane phospholipids in the acrosome reaction of guinea pig spermatozoa. J Exp Zool. (1984) 229:485–9. 10.1002/jez.1402290317 6423766

[105] Meizel S Turner KO. Stimulation of an exocytotic event, the hamster sperm acrosome reaction, by cis-unsaturated fatty acids. FEBS Lett. (1983) 161:315–8. 6617881 10.1016/0014-5793(83)81032-1

[106] Tavilani H Doosti M Abdi K Vaisiraygani A Joshaghani HR. Decreased polyunsaturated and increased saturated fatty acid concentration in spermatozoa from asthenozoospermic males as compared with normozoospermic males. Andrologia (2006) 38:173–8. 10.1111/j.1439-0272.2006.00735.x 16961570

[107] Andersen JM Rønning PO Herning H Bekken SD Haugen TB Witczak O. Fatty acid composition of spermatozoa is associated with BMI and with semen quality. Andrology (2016) 4:857–65. 10.1111/andr.12227 27371336

[108] Boerke A Brouwers JF Olkkonen VM van de Lest CHA Sostaric E Schoevers EJ. Involvement of bicarbonate-induced radical signaling in oxysterol formation and sterol depletion of capacitating mammalian sperm during *in vitro* fertilization. Biol Reprod. (2013) 88:21. 10.1095/biolreprod.112.101253 23115269

[109] Bielska AA Olsen BN Gale SE Mydock-McGrane L Krishnan K Baker NA. Side-chain oxysterols modulate cholesterol accessibility through membrane remodeling. Biochemistry (2014) 53:3042–51. 10.1021/bi5000096 24758724 PMC4020583

[110] Parks JE Lynch DV. Lipid composition and thermotropic phase behavior of boar, bull, stallion, and rooster sperm membranes. Cryobiology (1992) 29:255–66. 1582232 10.1016/0011-2240(92)90024-v

[111] Rejraji H Sion B Prensier G Carreras M Motta C Frenoux JM. Lipid remodeling of murine epididymosomes and spermatozoa during epididymal maturation. Biol Reprod. (2006) 74:1104–13. 10.1095/biolreprod.105.049304 16510839

[112] Whitfield M Pollet-Villard X Levy R Drevet JR Saez F. Posttesticular sperm maturation, infertility, and hypercholesterolemia. Asian J Androl. (2015) 17:742–8. 10.4103/1008-682X.155536 26067871 PMC4577583

[113] Jin SK Yang WX. Factors and pathways involved in capacitation: how are they regulated? Oncotarget (2017) 8:3600–27. 10.18632/oncotarget.12274 27690295 PMC5356907

[114] Jafaroghli M Abdi-Benemar H Zamiri MJ Khalili B Farshad A Shadparvar AA. Effects of dietary n-3 fatty acids and vitamin C on semen characteristics, lipid composition of sperm and blood metabolites in fat-tailed Moghani rams. Anim Reprod Sci. (2014) 147:17–24. 10.1016/j.anireprosci.2014.03.013 24745668

[115] Wazzan WC Gwatkin RB Thomas AJ. Zona drilling enhances fertilization by mouse caput epididymal sperm. Mol Reprod Dev. (1990) 27:332–6. 10.1002/mrd.1080270407 2264995

[116] Matsuo Y Nomata K Eguchi J Aoki D Hayashi T Hishikawa Y. Immunohistochemical analysis of connexin43 expression in infertile human testes. Acta Histochem Cytochem. (2007) 40:69–75. 10.1267/ahc.07001 17653298 PMC1931485

[117] Brehm R Marks A Rey R Kliesch S Bergmann M Steger K Altered expression of connexins 26 and 43 in Sertoli cells in seminiferous tubules infiltrated with carcinoma-*in-situ* or seminoma. J Pathol. (2002) 197:647–53. 10.1002/path.1140 12210085

[118] Segretain D Decrouy X Dompierre J Escalier D Rahman N Fiorini C. Sequestration of connexin43 in the early endosomes: an early event of Leydig cell tumor progression. Mol Carcinog. (2003) 38:179–87. 10.1002/mc.10160 14639657

[119] Boivin J Bunting L Collins JA Nygren KG. International estimates of infertility prevalence and treatment-seeking: potential need and demand for infertility medical care. Hum Reprod. (2007) 22:1506–12. 10.1093/humrep/dem046 17376819

[120] Jungwirth A Giwercman A Tournaye H Diemer T Kopa Z Dohle G European Association of Urology Working Group on Male Infertility. European Association of Urology guidelines on Male Infertility: the 2012 update. Eur Urol. (2012) 62:324–32. 10.1016/j.eururo.2012.04.048 22591628

[121] Dimopoulou C Goulis DG Corona G Maggi M. The complex association between metabolic syndrome and male hypogonadism. Metab Clin Exp. [Epub ahead of print]. (2018). 10.1016/j.metabol.2018.03.024 29656047

[122] Kalyani RR Dobs AS. Androgen deficiency, diabetes, and the metabolic syndrome in men. Curr Opin Endocrinol Diabetes Obes. (2007) 14:226–34. 10.1097/MED.0b013e32814db856 17940444

[123] Jensen TK Andersson AM Jørgensen N Andersen AG Carlsen E Petersen JH Skakkebaek NE. Body mass index in relation to semen quality and reproductive hormones among 1,558 Danish men. Fertil Steril. (2004) 82:863–70. 10.1016/j.fertnstert.2004.03.056 15482761

[124] Sermondade N Faure C Fezeu L Shayeb AG Bonde JP Jensen TK. BMI in relation to sperm count: an updated systematic review and collaborative meta-analysis. Hum Reprod Update (2013) 19:221–31. 10.1093/humupd/dms050 23242914 PMC3621293

[125] Samavat J Natali I Degl'Innocenti S Filimberti E Cantini G Di Franco A. Acrosome reaction is impaired in spermatozoa of obese men: a preliminary study. Fertil Steril. (2014) 102:1274–81.e2. 10.1016/j.fertnstert.2014.07.1248 25226854

[126] Dupont C Faure C Sermondade N Boubaya M Eustache F Clément P. Obesity leads to higher risk of sperm DNA damage in infertile patients. Asian J Androl. (2013) 15:622–5. 10.1038/aja.2013.65 23792341 PMC3881654

[127] Ramírez-Torres MA Carrera A Zambrana M. [High incidence of hyperestrogenemia and dyslipidemia in a group of infertile men]. Ginecol Obstet Mex. (2000) 68:224–9. 10902292

[128] Cross NL. Role of cholesterol in sperm capacitation. Biol Reprod. (1998) 59:7–11. 9674986 10.1095/biolreprod59.1.7

[129] Eisenberg ML Kim S Chen Z Sundaram R Schisterman EF Louis GMB. The relationship between male BMI and waist circumference on semen quality: data from the LIFE study. Hum Reprod. (2015) 30:493–4. 10.1093/humrep/deu322 25516559 PMC4303771

[130] Lu JC Jing J Yao Q Fan K Wang GH Feng RX. Relationship between Lipids Levels of Serum and Seminal Plasma and Semen Parameters in 631 Chinese Subfertile Men. PLoS ONE (2016) 11:e0146304. 10.1371/journal.pone.0146304 26726884 PMC4699695

[131] Filippi S Vignozzi L Morelli A Chavalmane AK Sarchielli E Fibbi B. Testosterone partially ameliorates metabolic profile and erectile responsiveness to PDE5 inhibitors in an animal model of male metabolic syndrome. J Sex Med. (2009) 6:3274–88. 10.1111/j.1743-6109.2009.01467.x 19732305

[132] Vignozzi L Morelli A Sarchielli E Comeglio P Filippi S Cellai I. Testosterone protects from metabolic syndrome-associated prostate inflammation: an experimental study in rabbit. J Endocrinol. (2012) 212:71–84. 10.1530/JOE-11-0289 22010203

[133] Maneschi E Vignozzi L Morelli A Mello T Filippi S Cellai I. FXR activation normalizes insulin sensitivity in visceral preadipocytes of a rabbit model of MetS. J Endocrinol. (2013) 218:215–31. 10.1530/JOE-13-0109 23750014

[134] Vignozzi L Morelli A Filippi S Comeglio P Chavalmane AK Marchetta M. Farnesoid X receptor activation improves erectile function in animal models of metabolic syndrome and diabetes. J. Sex Med. (2011) 8:57–77. 10.1111/j.1743-6109.2010.02073.x 20955313

[135] Maneschi E Morelli A Filippi S Cellai I Comeglio P Mazzanti B. Testosterone treatment improves metabolic syndrome-induced adipose tissue derangements. J Endocrinol. (2012) 215:347–62. 10.1530/JOE-12-0333 23045189

[136] Marchiani S Vignozzi L Filippi S Gurrieri B Comeglio P Morelli A. Metabolic syndrome-associated sperm alterations in an experimental rabbit model: relation with metabolic profile, testis and epididymis gene expression and effect of tamoxifen treatment. Mol Cell Endocrinol. (2015) 401:12–24. 10.1016/j.mce.2014.11.005 25451982

[137] Simón L Funes AK Yapur MA Cabrillana ME Monclus MA Boarelli PV. Manchette-acrosome disorders during spermiogenesis and low efficiency of seminiferous tubules in hypercholesterolemic rabbit model. PLoS ONE (2017) 12:e0172994. 10.1371/journal.pone.0172994 28241054 PMC5328279

[138] Morgan DH Ghribi O Hui L Geiger JD Chen X. Cholesterol-enriched diet disrupts the blood-testis barrier in rabbits. Am J Physiol Endocrinol Metab. (2014) 307:E1125–30. 10.1152/ajpendo.00416.2014 25336525 PMC4269676

[139] Fan Y Liu Y Xue K Gu G Fan W Xu Y. Diet-induced obesity in male C57BL/6 mice decreases fertility as a consequence of disrupted blood-testis barrier. PLoS ONE (2015) 10:e0120775. 10.1371/journal.pone.0120775 25886196 PMC4401673

[140] Ghanayem BI Bai R Kissling GE Travlos G Hoffler U. Diet-induced obesity in male mice is associated with reduced fertility and potentiation of acrylamide-induced reproductive toxicity. Biol Reprod. (2010) 82:96–104. 10.1095/biolreprod.109.078915 19696015 PMC2802115

[141] Bakos HW Mitchell M Setchell BP Lane M. The effect of paternal diet-induced obesity on sperm function and fertilization in a mouse model. Int J Androl. (2011) 34:402–10. 10.1111/j.1365-2605.2010.01092.x 20649934

[142] Fernandez CDB Bellentani FF Fernandes GSA Perobelli JE Favareto APA Nascimento AF. Diet-induced obesity in rats leads to a decrease in sperm motility. Reprod Biol Endocrinol. (2011) 9:32. 10.1186/1477-7827-9-32 21396114 PMC3068085

[143] Sánchez-Garrido MA Ruiz-Pino F Manfredi-Lozano M Leon S Garcia-Galiano D Castaño JP. Obesity-induced hypogonadism in the male: premature reproductive neuroendocrine senescence and contribution of Kiss1-mediated mechanisms. Endocrinology (2014) 155:1067–79. 10.1210/en.2013-1584 24424048

[144] Campos-Silva P Costa WS Sampaio FJB Gregorio BM. Prenatal and/or postnatal high-fat diet alters testicular parameters in adult Wistar Albino rats. Histol Histopathol. (2017) 33:407–16. 10.14670/HH-11-941 29083015

[145] Mu Y Yan WJ Yin TL Zhang Y Li J Yang J. Diet-induced obesity impairs spermatogenesis: a potential role for autophagy. Sci Rep. (2017) 7:43475. 10.1038/srep43475 28276438 PMC5343591

[146] Fullston T McPherson NO Owens JA Kang WX Sandeman LY Lane M. Paternal obesity induces metabolic and sperm disturbances in male offspring that are exacerbated by their exposure to an “obesogenic” diet. Physiol Rep. (2015) 3:12336. 10.14814/phy2.12336 25804263 PMC4393169

[147] Rodríguez-González GL Vega CC Boeck L Vázquez M Bautista CJ Reyes-Castro LA. Maternal obesity and overnutrition increase oxidative stress in male rat offspring reproductive system and decrease fertility. Int J Obes. (2015) 39:549–56. 10.1038/ijo.2014.209 25504042

[148] Vega A Martinot E Baptissart M De Haze A Vaz F Kulik W. Bile acid alters male mouse fertility in metabolic syndrome context. PLoS ONE (2015) 10:e0139946. 10.1371/journal.pone.0139946 26439743 PMC4595338

